# Initiating renal replacement therapy through incremental haemodialysis: Protocol for a randomized multicentre clinical trial

**DOI:** 10.1186/s13063-020-4058-0

**Published:** 2020-02-19

**Authors:** M. Fernández Lucas, G. Ruíz-Roso, J. L. Merino, R. Sánchez, H. Bouarich, J. A. Herrero, A. Muriel, J. Zamora, A. Collado

**Affiliations:** 1 Servicio de Nefrología, Hospital Universitario Ramón y Cajal, IRYCIS, Madrid, Spain; 2 Departamento de Medicina, Universidad de Alcala, Alcalá de Henares, Madrid, Spain; 3 Hospital Universitario del Henares, Madrid, Spain; 4 Hospital Universitario La Paz, Madrid, Spain; 5 Hospital Universitario Principe de Asturias, Alcalá de Henares, Madrid, Spain; 6 Hospital Universitario Clínico San Carlos, Madrid, Spain; 7 Unidad de Bioestadística, H. U, Ramón y Cajal, IRYCIS, Madrid, Spain

**Keywords:** Incremental Haemodialysis, Haemodialysis, Renal replacement therapy

## Abstract

**Background:**

Thrice-weekly haemodialysis is the usual dose when starting renal replacement therapy; however, this schedule is no longer appropriate since it does not consider residual renal function. Several reports have suggested the potential benefit of beginning haemodialysis less frequently and incrementally increasing the dose as the residual renal function decreases. However, all the data published so far are from observational studies. Thus, this clinical trial avoids any potential selection bias and will assess the possible benefits that have been observed in observational studies.

**Methods/design:**

This report describes the study protocol of a randomized prospective multi-centre open-label clinical trial to evaluate whether starting renal replacement therapy with twice-weekly haemodialysis sessions preserves residual renal function better than the standard thrice-weekly regimen. We also explore other clinical parameters, such as concentrations of uremic toxins, dialysis doses, control of anaemia, removal of medium-weight uremic toxins, nutritional status, quality of life, hospital admissions and mortality. Only incident haemodialysis patients who can maintain a urea clearance rate KrU ≥ 2.5 mL/min/1.73 m^2^ are eligible. Patient recruitment began on 1 January 2017 and will last for 2 years or until the required sample size has been recruited to ensure the established statistical power has been reached. The minimum follow-up period will be 1 year. Anuric patients with acute renal failure and patients who return to haemodialysis after a kidney transplant failure are excluded. It has been calculated that 44 patients should be recruited into each group to achieve a power of 80% in a two-sided comparison of means with a usual significance level of 0.05. A time-to-event analysis will estimate the probability of kidney function survival in both groups using the Kaplan–Meier method. Survival curves will be compared with log-rank tests. This survival analysis will be complemented with a proportional hazard model to estimate the hazard ratio of kidney function survival adjusted for any confounding factors. Analyses will be carried out in accordance with the intention-to-treat principle.

**Discussion:**

The incremental initiation of dialysis may preserve residual renal function better than the conventional treatment, with similar or higher survival rates, as reported by observational studies. To our knowledge, this is the first clinical trial to evaluate whether initiating renal replacement therapy with twice-weekly haemodialysis sessions preserves residual renal function better than beginning with the standard thrice-weekly regimen.

**Trial registration:**

ClinicalTrials.gov, NCT03302546. Registered on 5 October 2017.

## Background

The usual schedule is thrice-weekly haemodialysis sessions, and only a few patients receive dialysis less frequently [1]. Data from Spain indicate that whereas 8% of patients are dialysed more often than 3 days per week, only 1% do so at a lower frequency [1]. Based on model of urea kinetics, Gotch and Keen [2] proposed that an adequate dose of dialysis could be achieved with two dialysis sessions per week, as long as the residual urea clearance rate KrU ≥ 2.5 mL/min/1.73 m^2^. However, adherence to this scheme is low, based on data in the Dialysis Outcomes Practice Patterns Study [1] and on clinical guides, like the 2015 Kidney Disease Outcomes Quality Initiative (KDOQI) guidelines [3].

Periodically measuring the renal urea clearance rate is not common practice in haemodialysis units because renal function is considered to decrease rapidly after the onset of dialysis treatment. This could, in part, justify the poor implementation of lower-frequency haemodialysis. However, in recent years there has been growing interest in less frequent dialysis schemes [1, 4–8], though this is not without significant controversy [9–11].

Beginning haemodialysis less frequently and incrementally increasing the dose as residual renal function decreases is referred to as incremental or progressive haemodialysis [4–7, 11]. Most of the published works highlight that incremental haemodialysis may preserve residual kidney function [4–7], which has significant relevance for haemodialysis treatment [8, 12, 13]. Maintaining residual kidney function, other than allowing more liquid intake, contributes to a greater elimination of medium-sized molecules and has beneficial effects on anaemia, inflammation, and high blood pressure [8, 12–15]. The preservation of residual renal function is one of the adequacy goals of haemodialysis treatment in the KDOQI guidelines [3]. Moreover, incremental haemodialysis is associated with lower doses of erythropoietin [4, 7], better nutritional status [15, 16], lower concentrations of beta-2 microglobulin [7, 17], lower volume overload [7, 18], better quality of life [13], lower rate of hospital admissions [14, 19] and survival rates equal to or greater than those achieved with the conventional three-sessions per week scheme [7, 12, 16, 18–20].

The Haemodialysis Unit of the Nephrology Department of Hospital Universitario Ramón y Cajal is a pioneer in implementing an incremental haemodialysis program, having more than 10 years of experience [4, 5, 7, 17]. Our incremental program is based on the models of Gotch and Keen [2]. It uses a threshold of 2.5 mL/min/1.73 m^2^ to indicate when to increase the frequency of haemodialysis. This program has had satisfactory results and no clinical side effects in our previous experience [4, 5, 7, 17].

The percentage of patients who start renal replacement treatment with two weekly sessions has increased from 28% in 2006 to 84% in 2017. Currently, about 25% of the patients in our unit are treated with this twice-weekly frequency [17]. We have observed beneficial effects on the management of anaemia and hospitalization rates [3, 7], a lower concentration of beta-2 microglobulin (which is a marker of medium-sized molecules [7]) and no volume overload, as measured by bioimpedance [7]. The maintenance of residual renal function is similar among patients who start with an incremental haemodialysis regimen and those who begin peritoneal dialysis [5].

However, note that published studies on incremental haemodialysis are observational, with potential selection bias [4–7, 12, 15, 18]. In addition to differences in renal function at the start of haemodialysis, some centres exclude patients with certain comorbidities [11]. Thus, this clinical trial avoids selection bias and will assess the possible benefits that have been observed in observational studies.

To our knowledge, this is the first clinical trial to evaluate whether initiating renal replacement therapy with twice-weekly haemodialysis sessions preserves residual renal function better than beginning with the standard thrice-weekly regimen. Recently, the study protocol has been published of another clinical trial that assesses whether incrementally starting with one session of haemodialysis per week reduces mortality in incident patients compared with patients who start renal replacement therapy with the conventional method [21].

## Methods/design

### Hypothesis

Our hypothesis is that initiating renal replacement therapy with twice-weekly haemodialysis sessions preserves residual renal function better than the conventional regimen of beginning with thrice-weekly sessions. In addition, we hypothesise that maintaining renal function provides beneficial clinical effects, such as better control of anaemia, greater removal of medium-weight uremic toxins, and better nutritional status and quality of life, and has a safety profile like that of the conventional schedule.

### Outcomes

The main outcome is loss of kidney function, defined as urine volume output less than 100 mL in 24 h, 12 months after the initiation of haemodialysis therapy.

The secondary outcomes are: (1) erythropoietin dosage, (2) beta-2 microglobulin concentration, (3) hydration and nutritional status, (4) concentrations of uremic toxins, (5) dialysis dose, (6) number and duration of hospital admissions, (7) mortality and (8) quality of life.

In addition, cost-efficiency will be assessed by recording the cost of haemodialysis sessions and the monthly consumption of erythropoietin.

### Trial design

This is a randomized prospective multi-centre open-label clinical trial. Randomization will be stratified in every recruitment centre, ensuring a 1:1 ratio between the two study groups in all centres. The trial compares the initiation of renal replacement therapy with two haemodialysis sessions per week to the conventional three sessions per week in patients with residual urea clearance rate KrU ≥ 2.5 mL/min/1.73 m^2^. As treatment masking is not feasible, treatment allocation is centralized for all participating hospitals, and performed by an agent external to the researchers. Once a participating centre has verified that a patient meets the inclusion criteria, the randomization centre will provide the treatment group allocation. The investigators will not know the randomization sequence. The two groups will be those beginning with two haemodialysis sessions per week (2HD) and those beginning with three haemodialysis sessions per week (3HD).

### Participants

The participants are incident patients at five university hospitals in the Community of Madrid (Spain): H. U. Ramón y Cajal (coordinating centre), H. U. del Henares, H. U. La Paz, H. U. Príncipe de Asturias and H. U. Clínico San Carlos. The minimum follow-up period will be 1 year.

#### Inclusion criteria

Prospective participants must have stage 5 chronic kidney disease, must have started chronic haemodialysis and must be able to maintain a residual urea clearance rate KrU ≥ 2.5 mL/min/1.73 m^2^. Before enrolment, participants must sign the informed consent form.

#### Exclusion criteria

Anuric patients, patients with acute renal failure, and patients who return to haemodialysis after a kidney transplant failure will be excluded from the trial.

### Withdrawal criteria

Patients will be withdrawn from the study if they receive a renal transplant, die***,*** recover renal function or transfer to another facility or if they withdraw their informed consent.

### Assessments

The following will be assessed at the momento fo enrollment: age, sex, renal disease, Charlson comorbidity index, urine volume output in 24 h (mL), urea clearance (mL/min), creatinine clearance (mL/min).

The following will be assessed at the start of haemodialysis treatment (baseline) and every 2 months during a follow-up of at least 1 year: urine volume output in 24 h (mL), urea clearance (mL/min), creatinine clearance (mL/min), haemoglobin (g/dL), haematocrit (%), creatinine (mg/dL), urea (mg/dL), Na (mmol/L), K (mmol/L), albumin (g/dL), pre-albumin (mg/dL), calcium (mg/dL), phosphorus (mg/dL), parathyroid hormone (pg/mL), ferritin (ng/mL), beta-2 microglobulin (mg/L), brain-derived natriuretic peptide (pg/mL), C-reactive protein (mg/L), dose of dialysis (*Kt / V*), erythropoietin dosage (units per week per kg of weight), hydration, nutrition, hospital admissions, days of admission, causes of hospitalization and number of haemodialysis sessions.

The dose of dialysis is determined using the kinetic model of urea with the formulae modified from Gotch [22]. Dialysis *Kt* / *V* [3] is added to the residual renal function to give the total *Kt* / *V*:

Total *Kt* / *V* = dialysis *Kt* / *V* + (9.5 KrU) / *V* (for 2 haemodialysis sessions per week)

and

Total *Kt* / *V* = dialysis *Kt* / *V* + (5.5 KrU) / *V* (for 3 haemodialysis sessions per week),

where *V* is 58% of the dry weight (Watson formula) [23] and KrU is the residual urea clearance rate (mL/min).

Hydration and nutrition are measured by multi-frequency electrical bioimpedance (BCM®, Fresenius Medical Care). The following metrics are collected: overhydration (L), amount of extracellular water (L), amount of intracellular water (L), extracellular / intracellular ratio, body mass index (kg/m^2^), lean tissue index (kg/m^2^) and fat tissue index (kg/m^2^).

Quality of life will be assessed using the Kidney Disease Quality of Life Short Form at the start of haemodialysis treatment (baseline), at 6 months and at 12 months (close-out). This is a validated questionnaire for the haemodialysis population.

**Fig. 1:**
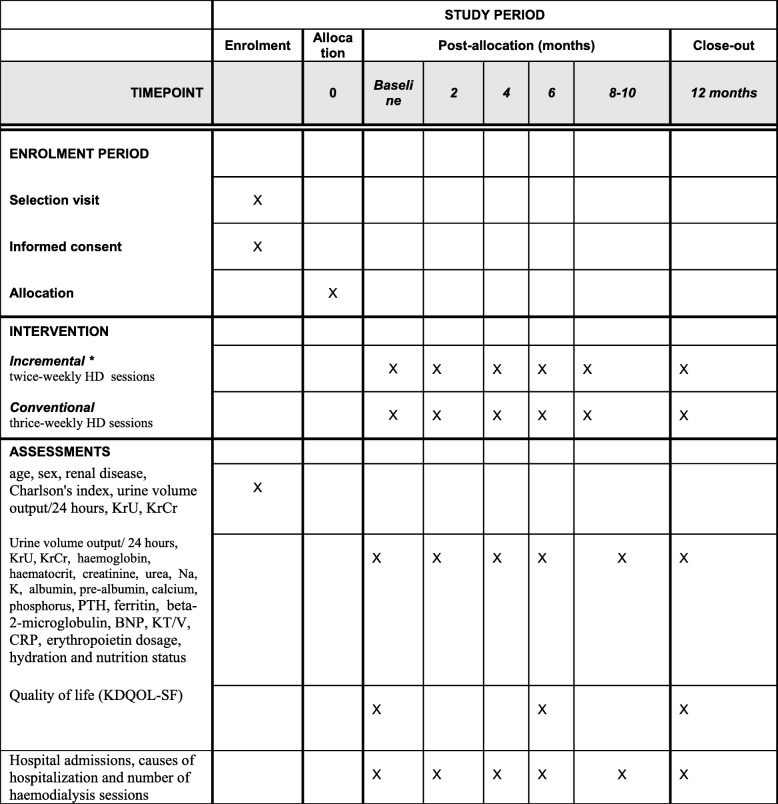
Schedule of enrolment, interventions and assessments * Patients receiving twice-weekly haemodialysis sessions are switched to the conventional arm if KrU < 2.5 mL/min/1.73 m^2^ in two determinations made in a 2-week interval, or if they develop uncontrolled hypertension, heart failure or a clinical event that indicates that the number of haemodialysis sessions should be increased. BNP brain-derived natriuretic peptide, CRP C-reactive protein, HD haemodialysis, KDQOL-SF Kidney Disease Quality of Life Short Form, KrCr creatinine clearance rate, KrU urea clearance rate, PTH parathyroid hormone

The schedule of enrolment, intervention and assessments is shown in Fig. 1.

### Criteria for progression

The dialysis technique used for each patient (high-flow haemodialysis or haemodiafiltration online) will be maintained throughout the follow-up period. The 3HD group will start with three sessions lasting 3–3.5 h to achieve an adequate dialysis *Kt* / *V*. The 2HD group will start with two sessions of 4 h.

According to Casino and López [24], to obtain an equivalent renal urea clearance rate of 11 mL/min, the adequate dialysis *Kt* / *V* should be 1.6 or 1.2 for 2 sessions per week and 3 sessions per week, respectively. Modifications to the dialysis procedure, such as increasing the blood flow, bath flow, dialyzer surface or duration of the dialysis session, will be made if patients do not reach an adequate *Kt* / *V*.

Participants in the 2HD group will be switched to the 3HD group if their urea clearance rate falls below 2.5 mL/min/1.73 m^2^ in two determinations made in a 2-week interval, or they develop uncontrolled hypertension, heart failure, or a clinical event that indicates that the number of haemodialysis sessions should be increased.

### Recruitment period

Patient recruitment began on 1 January 2017 and will be extended until the required sample size has been recruited to ensure the established statistical power has been reached. The minimum follow-up will be 1 year.

### Sample size

Considering the data published in observational studies [4, 5, 7] and assuming an average decrease in the glomerular filtration rate of 0.18 mL/min/month in the 2HD group and 0.33 mL/min/month in the 2HD group [7], with a standard deviation of 0.25, 44 patients should be recruited into each group to achieve a power of 80% in a two sided comparison of means with a usual significance level of 0.05.

### Data collection and statistical analysis

The data collected will be stored in a database specially designed for this study using Access (Microsoft Office Access 2007). The data will be entered by a dedicated member of the project team, who will ensure the integrity and quality of the data. The statistical analyses will be carried out with the statistics program Stata v.14 [25].

We will perform a blind interim analysis when at least half of the patients have been recruited and followed for at least 1 year. We will stop the trial if the group difference in the main outcome is statistically significant at a significance boundary *p* < 0.0051. The final analysis with the whole trial population will be carried out at the end of the follow-up at a significance level of 0.04795.

For the univariate analysis of quantitative variables, we will check the normality of the data using the Kolmogorov–Smirnov test. Depending on the assumption of normality, the two arms will be compared using parametric tests (Student’s *t*-test) or nonparametric tests (Mann–Whitney *U* test). The associations of categorical variables with the two treatment groups will be assessed by chi-squared tests or Fisher exact tests, as appropriate.

The loss of renal function is evaluated by the diuresis in 24 h at 12 months after initiation of haemodialysis therapy, and it is dichotomized with a threshold of 100 mL in 24 h. A time-to-event analysis will estimate the probability of loss of kidney function in both treatment groups using the Kaplan–Meier method. Survival curves will be compared with log-rank tests. This survival analysis will be complemented with a proportional hazard model to estimate the hazard ratio of kidney function survival adjusted for any confounding factors to identify any imbalance in the baseline characteristics of the patients.

All analyses will be carried out in accordance with the intention-to-treat and per-protocol principles, assuming that in the worst case the losses to follow-up are a failure of the allocated treatment arm. In the final analyses, for the comparisons between the two treatment groups, the probability of rejecting the null hypothesis will be adjusted according to the O’Brien and Fleming method [26].

### Limitations

Neither patients nor clinicians will be blind to the treatment modality. However, the main outcome in the study (anuria) is a quantitative and objective parameter. Other possible limitations may be due to insufficient recruitment such that the analysis population differs from the population treated in standard clinical practice.

## Discussion

The transition from pre-dialysis to renal replacement therapy is a crucial moment for patients with chronic renal failure and has significant clinical, social and occupational impacts [27]. Although dialysis therapy may predispose someone to losing their residual renal function, a significant proportion of dialysis patients maintain some degree of renal function long after the initiation of dialysis. In addition, maintaining the residual function may have a positive impact on the survival of haemodialysis patients [8, 12–15].

An incremental approach to the initiation of dialysis may allow some patients to retain more of their residual renal function with an improved quality of life and similar or higher survival rates than those receiving the conventional treatment, as reported by observational studies [4–7, 12, 16, 18, 19, 28]. We also consider that the incremental schedule facilitates the transition to renal replacement therapy and additionally, contributes to reducing health-care costs, not only because there are fewer haemodialysis sessions but also a lower consumption of erythropoietin [4, 7].

However, all previous studies have been observational, and prospective trials, such as this one, are required to determine, without selection bias, the potential benefits and safety of the incremental schedule [9–11, 28], and to establish the optimal group of patients who would benefit from this therapy.

This randomized clinical trial compares twice- versus thrice-weekly haemodialysis sessions for patients who can maintain a residual urea clearance rate KrU ≥ 2.5 mL/min/1.73 m^2^, without excluding a priori patients with severe comorbidities. In our experience, more than 80% of patients who started incremental renal replacement treatment did not suffer from any relevant complications [17]. For these patients, the dose of dialysis was progressively adjusted as their residual renal function declined. In our protocol, diuresis in 24 h is used as a proxy of residual renal function. The transition to more frequent treatment sessions occurs once residual renal function starts to decrease. However, it is essential to determine whether incremental haemodialysis is a safe form of dialysis treatment.

## Data Availability

Data sharing is not applicable to this article as no datasets were generated or analysed during the current study

## Abbreviations

2HD: Two haemodialysis sessions per week
3HD: Three haemodialysis sessions per week
BNP: Brain-derived natriuretic peptide
CRP: C-reactive protein
KDOQI: Kidney Disease Outcomes Quality Initiative
KDQOL-SF: Kidney Disease Quality of Life Short Form
KrCr: Creatinine clearance rate
KrU: Urea clearance rate
PTH: Parathyroid hormone
